# [2,6-Bis(6-methyl­quinolin-2-yl)pyridine-κ^3^ *N*,*N*′,*N*′′]dichloridomanganese(II)

**DOI:** 10.1107/S1600536810037037

**Published:** 2010-09-25

**Authors:** Xiao-Ping Li, Jian-She Zhao, Seik Weng Ng

**Affiliations:** a Department of Chemistry, Shaanxi Key Laboratory for Physico-Inorganic Chemistry, Northwest University, Xi’an 710069, People’s Republic of China; b Department of Chemistry, University of Malaya, 50603 Kuala Lumpur, Malaysia

## Abstract

In the mol­ecule of the title compound, [MnCl_2_(C_25_H_19_N_3_)], the three N atoms span the axial–equatorial–axial sites of the trigonal-bipyramidal coordination polyhedron; the geometry of the Mn^II^ atom is 34% distorted from trigonal-bipyramidal (towards square-pyramidal along the Berry pseudorotation pathway). One of the Cl atoms is disordered over two positions in a 0.82 (3):0.18 (3) ratio. Weak inter­molecular C—H⋯Cl hydrogen bonding occurs in the crystal structure.

## Related literature

For the synthesis of the *N*-heterocyclic ligand, see: Buu-Hoi *et al.* (1965 ▶). For a related structure, see: Li *et al.* (2010 ▶).
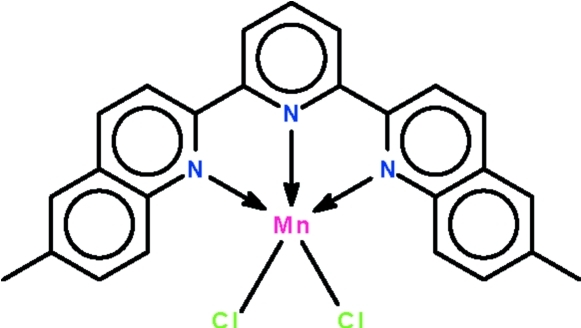


## Experimental

### 

#### Crystal data

[MnCl_2_(C_25_H_19_N_3_)]
*M* *_r_* = 487.27
Triclinic, 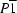

*a* = 9.6763 (8) Å
*b* = 10.3721 (9) Å
*c* = 10.5757 (9) Å
α = 95.099 (1)°
β = 97.499 (1)°
γ = 95.411 (1)°
*V* = 1042.13 (15) Å^3^
*Z* = 2
Mo *K*α radiation
μ = 0.91 mm^−1^
*T* = 100 K
0.30 × 0.10 × 0.05 mm

#### Data collection

Bruker SMART APEX diffractometer
Absorption correction: multi-scan (*SADABS*; Sheldrick, 1996 ▶) *T*_min_ = 0.772, *T*_max_ = 0.956
10000 measured reflections
4760 independent reflections
3704 reflections with *I* > 2σ(*I*)
*R*_int_ = 0.031

#### Refinement

*R*[*F* ^2^ > 2σ(*F* ^2^)] = 0.050
*wR*(*F* ^2^) = 0.151
*S* = 1.03
4760 reflections
292 parameters
7 restraints
H-atom parameters constrained
Δρ_max_ = 0.61 e Å^−3^
Δρ_min_ = −0.62 e Å^−3^

### 

Data collection: *APEX2* (Bruker, 2009 ▶); cell refinement: *SAINT* (Bruker, 2009 ▶); data reduction: *SAINT*; program(s) used to solve structure: *SHELXS97* (Sheldrick, 2008 ▶); program(s) used to refine structure: *SHELXL97* (Sheldrick, 2008 ▶); molecular graphics: *X-SEED* (Barbour, 2001 ▶); software used to prepare material for publication: *publCIF* (Westrip, 2010 ▶).

## Supplementary Material

Crystal structure: contains datablocks global, I. DOI: 10.1107/S1600536810037037/xu5031sup1.cif

Structure factors: contains datablocks I. DOI: 10.1107/S1600536810037037/xu5031Isup2.hkl

Additional supplementary materials:  crystallographic information; 3D view; checkCIF report

## Figures and Tables

**Table 1:** Selected bond lengths (Å)

Mn1—N1	2.305 (3)
Mn1—N2	2.186 (3)
Mn1—N3	2.332 (3)
Mn1—Cl1	2.3802 (17)
Mn1—Cl2	2.3375 (11)

**Table 2:** Hydrogen-bond geometry (Å, °)

*D*—H⋯*A*	*D*—H	H⋯*A*	*D*⋯*A*	*D*—H⋯*A*
C8—H8⋯Cl2^i^	0.95	2.64	3.486 (5)	149
C17—H17⋯Cl1^ii^	0.95	2.72	3.500 (9)	140

Symmetry codes: (i) 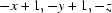; (ii) 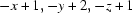.

**Fig. 1.**
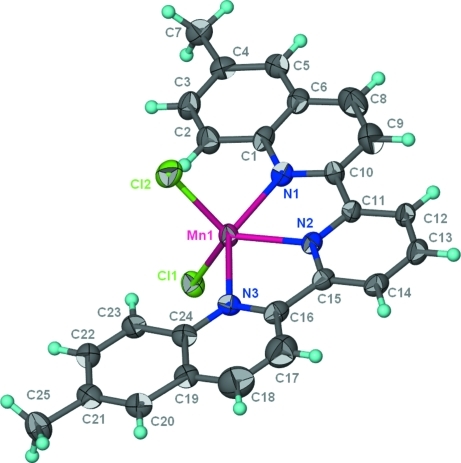
Thermal ellipsoid plot (Barbour, 2001) of MnCl2(C25H19N3) at the 70% probability level; hydrogen atoms are drawn as spheres of arbitrary radius. The disorder is not shown.

## supplementary crystallographic information

### Comment

A recent study reported the chromium(III) chloride adduct of 2,6-bis(*p*-bromphenylimino)pyridine; the *N*-heterocycle chelates to the metal atom in a terdentate manner (Li *et al.*, 2010). Bis[2'-(6-methylquinolinyl)]pyridine has a similar set of donor sites capable of binding in this manner, as demonstrated in the present manganese dichloride adduct (Scheme I, Fig. 1). In the molecule of MnCl_2_(C_25_H_19_N_3_), the three N atoms span the axial–equatorial-axial sites of the trigonal bipyramidal coordination polyhedron; the geometry of Fe is 34% distorted from the trigonal bipyramid along the Berry pseudorotation pathway. Intermolecular weak C—H···Cl hydrogen bonding occurs in the crystal structure (Table 2).

### Experimental

The ligand was synthesized by using a literature procedure (Buu-Hoi *et al.*, 1965).

Bis[2'-(6-methylquinolinyl)]pyridine (0.018 g, 0.05 mmol), and manganese chloride tetrahydrate (0.01 g, 0.05 mmol) along with five drops of 1 *M* hydrochloric acid were dissolved in ethanol (10 ml). The mixture was heated in a Teflon-lined, stainless-steel Parr bomb at 363 K for 120 h. The bomb was cooled at 5 K per hour. Deep orange crystals were isolated.

### Refinement

Carbon-bound H-atoms were placed in calculated positions (C—H 0.95–0.98 Å) and were included in the refinement in the riding model approximation, with *U*(H) set to 1.2–1.5*U*(C).

One of the chlorine atoms is disordered over two positions in a 82 (1):18 (1) ratio. The Mn–Cl pair of distances were restrained to within 0.01 Å of each other; the anisotropic temperature factors of the minor component were restrained to be nearly isotropic.

### Crystal data

**Table:**

[MnCl_2_(C_25_H_19_N_3_)]	*Z* = 2
*M**_r_* = 487.27	*F*(000) = 498
Triclinic, *P*1	*D*_x_ = 1.553 Mg m^−^^3^
Hall symbol: -P 1	Mo *K*α radiation, λ = 0.71073 Å
*a* = 9.6763 (8) Å	Cell parameters from 2981 reflections
*b* = 10.3721 (9) Å	θ = 2.7–28.1°
*c* = 10.5757 (9) Å	µ = 0.91 mm^−^^1^
α = 95.099 (1)°	*T* = 100 K
β = 97.499 (1)°	Prism, orange
γ = 95.411 (1)°	0.30 × 0.10 × 0.05 mm
*V* = 1042.13 (15) Å^3^	

### Data collection

**Table:**

Bruker SMART APEX diffractometer	4760 independent reflections
Radiation source: fine-focus sealed tube	3704 reflections with *I* > 2σ(*I*)
graphite	*R*_int_ = 0.031
ω scans	θ_max_ = 27.5°, θ_min_ = 2.0°
Absorption correction: multi-scan (*SADABS*; Sheldrick, 1996)	*h* = −12→12
*T*_min_ = 0.772, *T*_max_ = 0.956	*k* = −13→13
10000 measured reflections	*l* = −13→13

### Refinement

**Table:**

Refinement on *F*^2^	Primary atom site location: structure-invariant direct methods
Least-squares matrix: full	Secondary atom site location: difference Fourier map
*R*[*F*^2^ > 2σ(*F*^2^)] = 0.050	Hydrogen site location: inferred from neighbouring sites
*wR*(*F*^2^) = 0.151	H-atom parameters constrained
*S* = 1.03	*w* = 1/[σ^2^(*F*_o_^2^) + (0.0695*P*)^2^ + 1.8956*P*] where *P* = (*F*_o_^2^ + 2*F*_c_^2^)/3
4760 reflections	(Δ/σ)_max_ = 0.001
292 parameters	Δρ_max_ = 0.61 e Å^−^^3^
7 restraints	Δρ_min_ = −0.62 e Å^−^^3^

### Fractional atomic coordinates and isotropic or equivalent isotropic displacement parameters (Å^2^)

**Table:**

	*x*	*y*	*z*	*U*_iso_*/*U*_eq_	Occ. (<1)
Mn1	0.34023 (5)	0.80899 (5)	0.22288 (5)	0.02281 (16)	
Cl1	0.1607 (7)	0.7350 (2)	0.3414 (7)	0.0280 (8)	0.82 (3)
Cl1'	0.202 (4)	0.7316 (11)	0.378 (3)	0.033 (4)	0.18 (3)
Cl2	0.26785 (12)	0.84219 (10)	0.00968 (9)	0.0408 (3)	
N1	0.4349 (3)	0.6152 (3)	0.1893 (3)	0.0246 (6)	
N2	0.5637 (3)	0.8443 (3)	0.2966 (3)	0.0218 (6)	
N3	0.3883 (3)	1.0248 (3)	0.3127 (3)	0.0233 (6)	
C1	0.3623 (4)	0.5007 (3)	0.1294 (3)	0.0238 (7)	
C2	0.2180 (4)	0.4980 (4)	0.0882 (4)	0.0315 (8)	
H2	0.1724	0.5744	0.1012	0.038*	
C3	0.1431 (4)	0.3852 (3)	0.0292 (4)	0.0307 (8)	
H3	0.0455	0.3848	0.0020	0.037*	
C4	0.2061 (4)	0.2698 (3)	0.0075 (3)	0.0258 (7)	
C5	0.3468 (4)	0.2715 (3)	0.0468 (3)	0.0268 (7)	
H5	0.3910	0.1945	0.0326	0.032*	
C6	0.4273 (4)	0.3862 (3)	0.1081 (3)	0.0268 (7)	
C7	0.1162 (4)	0.1491 (3)	−0.0556 (4)	0.0300 (8)	
H7A	0.1753	0.0788	−0.0692	0.045*	
H7B	0.0676	0.1671	−0.1384	0.045*	
H7C	0.0470	0.1226	−0.0004	0.045*	
C8	0.5703 (5)	0.3949 (4)	0.1535 (4)	0.0449 (10)	
H8	0.6196	0.3205	0.1431	0.054*	
C9	0.6412 (5)	0.5111 (5)	0.2137 (4)	0.0452 (10)	
H9	0.7382	0.5156	0.2454	0.054*	
C10	0.5714 (3)	0.6197 (3)	0.2276 (3)	0.0218 (7)	
C11	0.6441 (3)	0.7459 (3)	0.2886 (3)	0.0234 (7)	
C12	0.7870 (3)	0.7643 (4)	0.3346 (3)	0.0250 (7)	
H12	0.8430	0.6941	0.3287	0.030*	
C13	0.8453 (4)	0.8864 (4)	0.3890 (3)	0.0294 (8)	
H13	0.9426	0.9013	0.4201	0.035*	
C14	0.7620 (4)	0.9875 (3)	0.3982 (3)	0.0268 (7)	
H14	0.8007	1.0719	0.4358	0.032*	
C15	0.6196 (3)	0.9620 (3)	0.3508 (3)	0.0224 (7)	
C16	0.5199 (4)	1.0619 (3)	0.3558 (3)	0.0257 (7)	
C17	0.5635 (5)	1.1919 (5)	0.4056 (5)	0.0507 (11)	
H17	0.6597	1.2182	0.4356	0.061*	
C18	0.4660 (5)	1.2819 (5)	0.4109 (5)	0.0514 (12)	
H18	0.4963	1.3696	0.4442	0.062*	
C19	0.3264 (4)	1.2453 (3)	0.3686 (3)	0.0262 (7)	
C20	0.2218 (4)	1.3312 (3)	0.3691 (3)	0.0281 (8)	
H20	0.2470	1.4196	0.4025	0.034*	
C21	0.0836 (4)	1.2892 (3)	0.3220 (3)	0.0288 (8)	
C22	0.0497 (4)	1.1579 (3)	0.2710 (3)	0.0273 (7)	
H22	−0.0448	1.1280	0.2370	0.033*	
C23	0.1494 (4)	1.0724 (3)	0.2691 (3)	0.0266 (7)	
H23	0.1230	0.9844	0.2349	0.032*	
C24	0.2906 (4)	1.1140 (3)	0.3175 (3)	0.0252 (7)	
C25	−0.0286 (4)	1.3793 (4)	0.3212 (4)	0.0363 (9)	
H25A	0.0144	1.4697	0.3304	0.055*	
H25B	−0.0816	1.3654	0.3926	0.055*	
H25C	−0.0920	1.3618	0.2400	0.055*	

### Atomic displacement parameters (Å^2^)

**Table:**

	*U*^11^	*U*^22^	*U*^33^	*U*^12^	*U*^13^	*U*^23^
Mn1	0.0177 (3)	0.0206 (3)	0.0285 (3)	0.00071 (19)	0.0000 (2)	−0.00009 (19)
Cl1	0.0207 (13)	0.0246 (6)	0.0377 (16)	−0.0016 (7)	0.0049 (13)	0.0008 (7)
Cl1'	0.026 (7)	0.031 (3)	0.040 (6)	−0.003 (3)	0.008 (6)	−0.003 (3)
Cl2	0.0500 (6)	0.0386 (5)	0.0323 (5)	0.0163 (4)	−0.0063 (4)	0.0018 (4)
N1	0.0245 (15)	0.0239 (14)	0.0248 (14)	0.0002 (11)	0.0016 (12)	0.0044 (11)
N2	0.0199 (14)	0.0219 (14)	0.0222 (13)	−0.0013 (11)	0.0007 (11)	0.0013 (10)
N3	0.0243 (15)	0.0230 (14)	0.0230 (14)	0.0021 (11)	0.0056 (11)	0.0013 (11)
C1	0.0210 (17)	0.0246 (16)	0.0240 (16)	−0.0048 (13)	−0.0002 (13)	0.0051 (13)
C2	0.0276 (19)	0.0251 (18)	0.040 (2)	0.0040 (14)	0.0006 (16)	0.0013 (15)
C3	0.0240 (18)	0.0284 (18)	0.036 (2)	−0.0020 (14)	−0.0037 (15)	0.0017 (15)
C4	0.0324 (19)	0.0213 (16)	0.0234 (16)	−0.0006 (14)	0.0044 (14)	0.0035 (13)
C5	0.0303 (19)	0.0253 (17)	0.0259 (17)	0.0047 (14)	0.0050 (14)	0.0062 (13)
C6	0.0251 (18)	0.0309 (18)	0.0246 (17)	0.0016 (14)	0.0022 (14)	0.0077 (14)
C7	0.033 (2)	0.0240 (17)	0.0318 (19)	−0.0015 (15)	0.0034 (15)	0.0036 (14)
C8	0.045 (3)	0.039 (2)	0.052 (3)	0.0141 (19)	0.009 (2)	0.0015 (19)
C9	0.032 (2)	0.054 (3)	0.048 (2)	0.0101 (19)	0.0015 (19)	0.000 (2)
C10	0.0163 (15)	0.0232 (16)	0.0257 (16)	−0.0003 (12)	0.0036 (13)	0.0032 (13)
C11	0.0203 (16)	0.0299 (18)	0.0200 (15)	0.0047 (13)	0.0012 (13)	0.0035 (13)
C12	0.0174 (16)	0.0330 (18)	0.0236 (16)	0.0010 (13)	0.0008 (13)	0.0017 (14)
C13	0.0174 (17)	0.045 (2)	0.0247 (17)	−0.0012 (15)	0.0012 (13)	0.0034 (15)
C14	0.0236 (18)	0.0297 (18)	0.0241 (16)	−0.0066 (14)	0.0020 (13)	−0.0022 (14)
C15	0.0206 (16)	0.0256 (17)	0.0198 (15)	−0.0034 (13)	0.0030 (12)	0.0019 (12)
C16	0.0261 (18)	0.0268 (17)	0.0225 (16)	−0.0050 (14)	0.0030 (13)	0.0017 (13)
C17	0.047 (3)	0.055 (3)	0.047 (3)	0.000 (2)	0.002 (2)	0.003 (2)
C18	0.060 (3)	0.041 (2)	0.051 (3)	−0.001 (2)	0.007 (2)	0.002 (2)
C19	0.0278 (18)	0.0258 (17)	0.0253 (17)	0.0009 (14)	0.0050 (14)	0.0042 (13)
C20	0.034 (2)	0.0226 (17)	0.0284 (18)	0.0020 (14)	0.0062 (15)	0.0050 (13)
C21	0.033 (2)	0.0279 (18)	0.0280 (17)	0.0068 (15)	0.0084 (15)	0.0079 (14)
C22	0.0265 (18)	0.0276 (18)	0.0280 (17)	0.0024 (14)	0.0039 (14)	0.0045 (14)
C23	0.0272 (18)	0.0220 (16)	0.0294 (17)	0.0005 (14)	0.0030 (14)	−0.0002 (13)
C24	0.0285 (18)	0.0251 (17)	0.0231 (16)	0.0018 (14)	0.0064 (14)	0.0053 (13)
C25	0.034 (2)	0.0282 (19)	0.048 (2)	0.0066 (16)	0.0070 (18)	0.0060 (17)

### Geometric parameters (Å, °)

**Table:**

Mn1—N1	2.305 (3)	C9—H9	0.9500
Mn1—N2	2.186 (3)	C10—C11	1.477 (5)
Mn1—N3	2.332 (3)	C11—C12	1.393 (5)
Mn1—Cl1	2.3802 (17)	C12—C13	1.381 (5)
Mn1—Cl1'	2.388 (7)	C12—H12	0.9500
Mn1—Cl2	2.3375 (11)	C13—C14	1.385 (5)
N1—C10	1.325 (4)	C13—H13	0.9500
N1—C1	1.378 (4)	C14—C15	1.395 (5)
N2—C15	1.335 (4)	C14—H14	0.9500
N2—C11	1.344 (4)	C15—C16	1.483 (5)
N3—C16	1.305 (4)	C16—C17	1.408 (6)
N3—C24	1.387 (5)	C17—C18	1.391 (7)
C1—C2	1.405 (5)	C17—H17	0.9500
C1—C6	1.410 (5)	C18—C19	1.373 (6)
C2—C3	1.370 (5)	C18—H18	0.9500
C2—H2	0.9500	C19—C20	1.410 (5)
C3—C4	1.408 (5)	C19—C24	1.413 (5)
C3—H3	0.9500	C20—C21	1.380 (5)
C4—C5	1.369 (5)	C20—H20	0.9500
C4—C7	1.505 (5)	C21—C22	1.411 (5)
C5—C6	1.414 (5)	C21—C25	1.498 (5)
C5—H5	0.9500	C22—C23	1.372 (5)
C6—C8	1.396 (6)	C22—H22	0.9500
C7—H7A	0.9800	C23—C24	1.407 (5)
C7—H7B	0.9800	C23—H23	0.9500
C7—H7C	0.9800	C25—H25A	0.9800
C8—C9	1.386 (6)	C25—H25B	0.9800
C8—H8	0.9500	C25—H25C	0.9800
C9—C10	1.373 (6)		
			
N2—Mn1—N1	72.62 (10)	C8—C9—H9	119.9
N2—Mn1—N3	71.80 (10)	N1—C10—C9	121.3 (3)
N1—Mn1—N3	144.34 (10)	N1—C10—C11	117.0 (3)
N2—Mn1—Cl2	118.47 (8)	C9—C10—C11	121.6 (3)
N1—Mn1—Cl2	99.09 (8)	N2—C11—C12	121.2 (3)
N3—Mn1—Cl2	99.72 (8)	N2—C11—C10	115.7 (3)
N2—Mn1—Cl1	125.3 (2)	C12—C11—C10	123.1 (3)
N1—Mn1—Cl1	98.25 (11)	C13—C12—C11	118.6 (3)
N3—Mn1—Cl1	100.18 (10)	C13—C12—H12	120.7
Cl2—Mn1—Cl1	116.2 (2)	C11—C12—H12	120.7
N2—Mn1—Cl1'	112.7 (11)	C12—C13—C14	120.1 (3)
N1—Mn1—Cl1'	93.8 (5)	C12—C13—H13	120.0
N3—Mn1—Cl1'	97.6 (3)	C14—C13—H13	120.0
Cl2—Mn1—Cl1'	128.8 (11)	C13—C14—C15	118.2 (3)
Cl1—Mn1—Cl1'	12.6 (9)	C13—C14—H14	120.9
C10—N1—C1	119.5 (3)	C15—C14—H14	120.9
C10—N1—Mn1	115.3 (2)	N2—C15—C14	121.6 (3)
C1—N1—Mn1	125.2 (2)	N2—C15—C16	115.2 (3)
C15—N2—C11	120.2 (3)	C14—C15—C16	123.2 (3)
C15—N2—Mn1	120.4 (2)	N3—C16—C17	120.5 (4)
C11—N2—Mn1	119.4 (2)	N3—C16—C15	117.4 (3)
C16—N3—C24	119.6 (3)	C17—C16—C15	122.1 (3)
C16—N3—Mn1	115.1 (2)	C18—C17—C16	120.2 (4)
C24—N3—Mn1	125.2 (2)	C18—C17—H17	119.9
N1—C1—C2	118.7 (3)	C16—C17—H17	119.9
N1—C1—C6	122.5 (3)	C19—C18—C17	120.6 (4)
C2—C1—C6	118.8 (3)	C19—C18—H18	119.7
C3—C2—C1	119.9 (3)	C17—C18—H18	119.7
C3—C2—H2	120.1	C18—C19—C20	123.9 (4)
C1—C2—H2	120.1	C18—C19—C24	116.1 (4)
C2—C3—C4	122.1 (3)	C20—C19—C24	120.0 (3)
C2—C3—H3	118.9	C21—C20—C19	121.2 (3)
C4—C3—H3	118.9	C21—C20—H20	119.4
C5—C4—C3	118.5 (3)	C19—C20—H20	119.4
C5—C4—C7	122.5 (3)	C20—C21—C22	118.1 (3)
C3—C4—C7	118.9 (3)	C20—C21—C25	121.9 (3)
C4—C5—C6	120.9 (3)	C22—C21—C25	120.0 (3)
C4—C5—H5	119.6	C23—C22—C21	121.8 (3)
C6—C5—H5	119.6	C23—C22—H22	119.1
C8—C6—C1	115.9 (3)	C21—C22—H22	119.1
C8—C6—C5	124.3 (4)	C22—C23—C24	120.5 (3)
C1—C6—C5	119.8 (3)	C22—C23—H23	119.8
C4—C7—H7A	109.5	C24—C23—H23	119.8
C4—C7—H7B	109.5	N3—C24—C23	118.7 (3)
H7A—C7—H7B	109.5	N3—C24—C19	122.9 (3)
C4—C7—H7C	109.5	C23—C24—C19	118.4 (3)
H7A—C7—H7C	109.5	C21—C25—H25A	109.5
H7B—C7—H7C	109.5	C21—C25—H25B	109.5
C9—C8—C6	120.6 (4)	H25A—C25—H25B	109.5
C9—C8—H8	119.7	C21—C25—H25C	109.5
C6—C8—H8	119.7	H25A—C25—H25C	109.5
C10—C9—C8	120.1 (4)	H25B—C25—H25C	109.5
C10—C9—H9	119.9		
			
N2—Mn1—N1—C10	−0.7 (2)	C1—N1—C10—C11	178.6 (3)
N3—Mn1—N1—C10	−4.7 (3)	Mn1—N1—C10—C11	1.2 (4)
Cl2—Mn1—N1—C10	116.4 (2)	C8—C9—C10—N1	2.9 (6)
Cl1—Mn1—N1—C10	−125.2 (3)	C8—C9—C10—C11	−178.6 (4)
Cl1'—Mn1—N1—C10	−113.3 (10)	C15—N2—C11—C12	−0.8 (5)
N2—Mn1—N1—C1	−177.9 (3)	Mn1—N2—C11—C12	−179.9 (2)
N3—Mn1—N1—C1	178.1 (2)	C15—N2—C11—C10	179.6 (3)
Cl2—Mn1—N1—C1	−60.8 (3)	Mn1—N2—C11—C10	0.5 (4)
Cl1—Mn1—N1—C1	57.5 (3)	N1—C10—C11—N2	−1.2 (4)
Cl1'—Mn1—N1—C1	69.4 (10)	C9—C10—C11—N2	−179.8 (3)
N1—Mn1—N2—C15	−179.0 (3)	N1—C10—C11—C12	179.2 (3)
N3—Mn1—N2—C15	−1.5 (2)	C9—C10—C11—C12	0.6 (5)
Cl2—Mn1—N2—C15	89.9 (2)	N2—C11—C12—C13	−0.1 (5)
Cl1—Mn1—N2—C15	−91.2 (3)	C10—C11—C12—C13	179.4 (3)
Cl1'—Mn1—N2—C15	−92.3 (4)	C11—C12—C13—C14	0.7 (5)
N1—Mn1—N2—C11	0.1 (2)	C12—C13—C14—C15	−0.3 (5)
N3—Mn1—N2—C11	177.6 (3)	C11—N2—C15—C14	1.1 (5)
Cl2—Mn1—N2—C11	−91.0 (2)	Mn1—N2—C15—C14	−179.7 (2)
Cl1—Mn1—N2—C11	87.9 (3)	C11—N2—C15—C16	−179.1 (3)
Cl1'—Mn1—N2—C11	86.8 (4)	Mn1—N2—C15—C16	0.0 (4)
N2—Mn1—N3—C16	3.0 (2)	C13—C14—C15—N2	−0.6 (5)
N1—Mn1—N3—C16	6.9 (3)	C13—C14—C15—C16	179.7 (3)
Cl2—Mn1—N3—C16	−114.0 (2)	C24—N3—C16—C17	−0.8 (5)
Cl1—Mn1—N3—C16	127.0 (3)	Mn1—N3—C16—C17	176.4 (3)
Cl1'—Mn1—N3—C16	114.4 (11)	C24—N3—C16—C15	178.8 (3)
N2—Mn1—N3—C24	180.0 (3)	Mn1—N3—C16—C15	−4.0 (4)
N1—Mn1—N3—C24	−176.0 (2)	N2—C15—C16—N3	2.8 (4)
Cl2—Mn1—N3—C24	63.1 (3)	C14—C15—C16—N3	−177.4 (3)
Cl1—Mn1—N3—C24	−56.0 (3)	N2—C15—C16—C17	−177.6 (3)
Cl1'—Mn1—N3—C24	−68.5 (11)	C14—C15—C16—C17	2.2 (5)
C10—N1—C1—C2	−179.3 (3)	N3—C16—C17—C18	0.9 (6)
Mn1—N1—C1—C2	−2.2 (4)	C15—C16—C17—C18	−178.7 (4)
C10—N1—C1—C6	0.8 (5)	C16—C17—C18—C19	0.4 (7)
Mn1—N1—C1—C6	177.9 (2)	C17—C18—C19—C20	−179.0 (4)
N1—C1—C2—C3	−179.5 (3)	C17—C18—C19—C24	−1.6 (6)
C6—C1—C2—C3	0.3 (5)	C18—C19—C20—C21	178.2 (4)
C1—C2—C3—C4	−0.1 (6)	C24—C19—C20—C21	0.9 (5)
C2—C3—C4—C5	−0.2 (6)	C19—C20—C21—C22	−1.1 (5)
C2—C3—C4—C7	178.8 (3)	C19—C20—C21—C25	−179.6 (3)
C3—C4—C5—C6	0.2 (5)	C20—C21—C22—C23	1.0 (5)
C7—C4—C5—C6	−178.7 (3)	C25—C21—C22—C23	179.6 (3)
N1—C1—C6—C8	1.0 (5)	C21—C22—C23—C24	−0.7 (5)
C2—C1—C6—C8	−178.8 (4)	C16—N3—C24—C23	179.3 (3)
N1—C1—C6—C5	179.6 (3)	Mn1—N3—C24—C23	2.4 (4)
C2—C1—C6—C5	−0.3 (5)	C16—N3—C24—C19	−0.5 (5)
C4—C5—C6—C8	178.4 (4)	Mn1—N3—C24—C19	−177.4 (2)
C4—C5—C6—C1	0.0 (5)	C22—C23—C24—N3	−179.3 (3)
C1—C6—C8—C9	−0.9 (6)	C22—C23—C24—C19	0.4 (5)
C5—C6—C8—C9	−179.4 (4)	C18—C19—C24—N3	1.7 (5)
C6—C8—C9—C10	−0.9 (7)	C20—C19—C24—N3	179.2 (3)
C1—N1—C10—C9	−2.8 (5)	C18—C19—C24—C23	−178.1 (4)
Mn1—N1—C10—C9	179.8 (3)	C20—C19—C24—C23	−0.5 (5)

### Hydrogen-bond geometry (Å, °)

**Table:**

*D*—H···*A*	*D*—H	H···*A*	*D*···*A*	*D*—H···*A*
C8—H8···Cl2^i^	0.95	2.64	3.486 (5)	149
C17—H17···Cl1^ii^	0.95	2.72	3.500 (9)	140

Symmetry codes: (i) −*x*+1, −*y*+1, −*z*; (ii) −*x*+1, −*y*+2, −*z*+1.
